# DHA inhibits invasion and metastasis in NSCLC cells by interfering with CCL18/STAT3 signaling pathway

**DOI:** 10.1007/s10238-022-00906-0

**Published:** 2022-10-10

**Authors:** Hai-qing Luo, Yu-meng Huang, Jing Li, Xu-dong Tang, Ran Chen, Yan Wang, Jing Ren, Qiu-qin Dai, Liu-bo Lan, Jiang-yan Chen, Xiang-yong Li

**Affiliations:** 1 Center of Oncology, The Affiliated Hospital of Guangdong Medical University, Zhanjiang, 524001 People’s Republic of China; 2 Key Laboratory for Biologically Active Molecular of Department of Education of Guangdong Province, Guangdong Medical University, Zhanjiang, 524023 People’s Republic of China; 3 Institute of Biochemistry and Molecular Biology, Guangdong Medical University, Zhanjiang, 524023 People’s Republic of China

**Keywords:** Non-small cell lung cancer, Omega-3, CCL18, Metastasis, Invasion

## Abstract

Omega-3 has been proposed as an antitumor substance that suppresses the growth and metastasis of multiple types of tumor cells, including lung cancer, but the specific mechanisms involved remain obscure. Our previous studies showed that the expression of chemokine ligand 18 was related to the migration and metastasis of non-small cell lung cancer. Here, we aim to explore whether omega-3 inhibits invasion and metastasis of NSCLC by regulating the expression of CCL18. The expression of CCL18, metastasis- and epithelial-mesenchymal transition (EMT)-related genes at mRNA and protein levels in NSCLC cell lines were detected by RT-qPCR and Western blot, respectively. The metastatic and invasive capability of NSCLC cells were evaluated by scratch wound healing and Transwell assays, respectively. Our results showed that the level of CCL18 is positively associated with metastatic ability of NSCLC cells. Docosahexaenoic acid, an important long-chain, polyunsaturated omega-3 (*n*−3) fatty acid, significantly inhibited invasion and metastasis of NSCLC cells, and concomitantly downregulated the expression of metastasis- and EMT-related genes and p-STAT3 signaling pathway. Additionally, we found that DHA inhibited CCL18 expression in lung cancer cells, while overexpression of CCL18 effectively reversed DHA-mediated downregulation in the expression of metastasis- and EMT-related genes and p-STAT3 signaling as well as DHA-mediated inhibitory effect on metastasis and invasion of NSCLC cells. DHA inhibits NSCLC cell invasion and metastasis possibly through targeted inhibition of CCL18/ STAT3 signaling pathway and EMT process.

**Supplementary Information:**

The online version contains supplementary material available at 10.1007/s10238-022-00906-0.

## Introduction

Lung cancer is the most common malignant tumor with a high mortality rate in the world [1]. Non-small cell lung cancer (NSCLC) accounts for about 80% to 85% of all lung cancers [2–4]. Lung cancer is characterized by its aggressive behaviors, including the high rate of local and distant metastasis and recurrence [5, 6], among which the metastasis rate of NSCLC is as high as 47.3% [7]. Even though much progress has been made in our understanding of the mechanisms underlying the pathogenesis of NSCLC, less is known about the molecular mechanisms for metastasis and recurrence of NSCLC, and development of novel therapeutic approaches for an effective control of metastatic disease will significantly benefit patients with this fatal malignancy.

The tumor microenvironment (TME) is a complex ecosystem composed of a milieu of cellular and noncellular components, including various types of immune cells and stromal cells (e.g. cancer-associated fibroblasts and endothelial cells) and their secreted cytokines, chemokines, growth factors, and other bioactive mediators, which play an important role in the development, invasion, and metastasis of tumors [8]. Tumor-associated macrophages (TAMs), the most abundant immune cells in TME, can be classified into anti-tumoral M1-like and pro-tumoral M2-like phenotypes [9]. A wealth body of evidence has shown that TAMs play critical roles in multiple hallmarks of cancer, such as aberrant survival and proliferative ability, angiogenesis, invasion/metastasis, recurrence, and immune evasion of cancer cells, and are also closely correlated with a poor prognosis in various types of tumors [10]. CC chemokine ligand 18 (CCL18), an important chemotactic cytokine, is mainly derived from M2-like TAMs [11] and plays a critical role in tumorigenesis and progression.

Omega-3 polyunsaturated fatty acids are the human essential polyunsaturated fatty acids and play a role in both human health and diseases [12]. Docosahexaenoic acid (DHA) as the main components of omega-3 has been demonstrated to have beneficial effects on various types of cancer due to its potent inhibitor effect on tumor growth, invasion, and metastasis [13]. Interestingly, DHA has been shown to be involved in the reprogramming of TME through its anti-inflammatory activity [14]. Our previous studies showed that CCL18 is highly expressed in NSCLC tissues and the serum of lung cancer patients, whereby its expression levels were positively correlated with a late clinical stage and lymph node metastasis of NSCLC [15]. Importantly, we also found that the CCL18 levels were significantly reduced in NSCLC patients treated with omega-3 [16]. In the present study, we aim to further explore whether DHA inhibits the invasion and metastasis of NSCLC cells by downregulating the expression of CCL18 and the related signaling pathways.

## Materials and methods

### Cell culture

The NSCLC line A459, H460 and human normal lung epithelial cell line BEAS-2B were preserved by this research group, while 95C and 95D cells were purchased from the Cell Bank of the Chinese Academy of Sciences. The above cells were cultured in RPMI 1640 (Gibco, U.S.) medium supplemented with 10% fetal bovine serum (FBS) (Corning, New York, USA) and 1% penicillin streptomycin solution. All cell lines were cultured in a humidified incubator at 37 °C with 5% CO2. Cells at the logarithmic growth phase were used for experiments.

### CCK8 assay

Cells were seeded into 96-well plate at a cell density of 5 × 103/well. DHA (NU-CHEK, USA) was added at different concentrations, and cell proliferation was measured after 24 h, 48 h, and 72 h. Detected cell proliferation at 24 h, 48 h, and 72 h, respectively. 10 µL CCK8 reagent was added to wells 1–4 h for CCK8 detection. The absorbance at 450 nm was measured with a microplate reader (Thermo Fisher Scientific, Madrid, Spain). The number of viable cells was positively correlated with the OD value at 450 nm. All assays were performed in triplicate.

### Wound healing assay

To analyze the effect of DHA on the invasive and metastatic ability of lung cancer cells, A549 and 95D cells were treated with DHA for 48 h. PBS was added to the confluent monolayers for three rinses after scratching with a 20 µL pipette tip. The wound width was observed using a microscope at 0 h, 12 h, and 24 h after scratching, and the wound areas were analyzed with image J.

### Transwell migration and invasion assay

In transwell migration experiment, cells were resuspended in serum-free medium at a density of 1 × 10^5^/mL. Then, 200 µL of cell suspension were added into the upper chamber and 500 µL of medium containing 10% FBS into the lower chamber and incubated for 12 h in the presence or absence of DHA. Afterward, cells were wiped off from the membrane in the upper chamber with cotton swabs and stained with crystal violet for 30 min at 37 °C. Next, the cells were observed and photographed under a microscope (Leica, Germany). The integrated density of the protein bands was measured with Image J. In invasion experiment, Matrigel (Corning, USA) was diluted with serum-free medium at an 8:1 ratio and added into the upper chamber to form an artificial basement membrane. Then, the experiment was performed similarly as the cell migration assay as described above.

### Real-time qPCR (RT-qPCR)

Trizol regent (Life Technology, USA) was used to extract the total RNA, and the reverse transcription experiment was performed according to the instructions of PrimeScriptTM RT reagent Kit with gDNA Eraser (Perfect Real Time) (TaKaRa, China). We quantified the mRNA of differential genes with SYBR^®^ Premix Ex TaqTM II (Tli RNaseH Plus) (TaKaRa, China) using the operating method of Applied Biosystems^®^ 7500 Real-Time PCR Systems (Thermo Fisher SCIENTIFIC, USA). All primers used for RT-qPCR are shown in Table 1. The relative expression of mRNA was calculated by the 2-ΔΔC*t* method. *β*-actin was used as the reference gene for mRNA.

**Table 1:** Primer sequences

Gene	Primer (5′ → 3′)	Length (bp)
MMP1	F: CTCTGGAGTAATGTCACACCTCT	199
	R: TGTTGGTCCACCTTTCATCTTC	
MMP2	F: GATACCCCTTTGACGGTAAGGA	112
	R: CCTTCTCCCAAGGTCCATAGC	
MMP7	F: GAGTGAGCTACAGTGGGAACA	158
	R: CTATGACGCGGGAGTTTAACAT	
MMP9	F: GGGACGCAGACATCGTCATC	139
	R: TCGTCATCGTCGAAATGGGC	
Snail	F: ACTGCAACAAGGAATACCTCAG	242
	R: GCACTGGTACTTCTTGACATCTG	
Slug	F: TGTGACAAGGAATATGTGAGCC	203
	R: TGAGCCCTCAGATTTGACCTG	
Vimentin	F: TGCCGTTGAAGCTGCTAACTA	248
	R: CCAGAGGGAGTGAATCCAGATTA	
*β*-catenin	F: CATCTACACAGTTTGATGCTGCT	150
	R: GCAGTTTTGTCAGTTCAGGGA	
N-cadherin	F: AGCCAACCTTAACTGAGGAGT	136
	R: GGCAAGTTGATTGGAGGGATG	
Zeb1	F: CAGCTTGATACCTGTGAATGGG	106
	R: TATCTGTGGTCGTGTGGGACT	
*β*-actin	F: CATGTACGTTGCTATCCAGGC	250
	R: CTCCTTAATGTCACGCACGAT	

### Western blot

After treating with DHA for 48 h, cells were washed with PBS 3 times. We extracted total cell protein lysate using cell lysis buffer (Beyotime Biotechnology, China) containing protease inhibitors (Beyotime Biotechnology, China) and protein phosphatase inhibitors (Roche, Basel, Switzerland). The BAC method was used to determine the protein concentration of each sample. Denatured protein samples were separated on the SDS-PAGE gel and transferred to polyvinylidene difluoride membranes (PVDF) membrane (Millipore, USA). PVDF membranes were blocked with 5% nonfat milk at room temperature for 2 h. After rinsing with TBST, the membranes were incubated with primary antibodies overnight at 4 °C. TBST rinsed membranes 3 times and incubated with secondary antibodies for 2 h. The protein bands were visualized using ECL chemiluminescence detection reagents (Beyotime Biotechnology, China) and analyzed by Image J software.

### Statistical analysis

All experiments were conducted at least three independent experiments with at least three technical repetitions. SPSS 19.0 statistical software package was used to analyze all data. Quantitative data were expressed by mean ± standard deviation (SD). One-way analysis of variance was applied for multiple groups, and the least-significant difference (LSD) was used for pairwise comparisons between groups when the variance was homogeneous or the Dunnett's T3 was applied.

## Results

### Expression of CCL18 correlates with metastasis

**Fig. 1:**
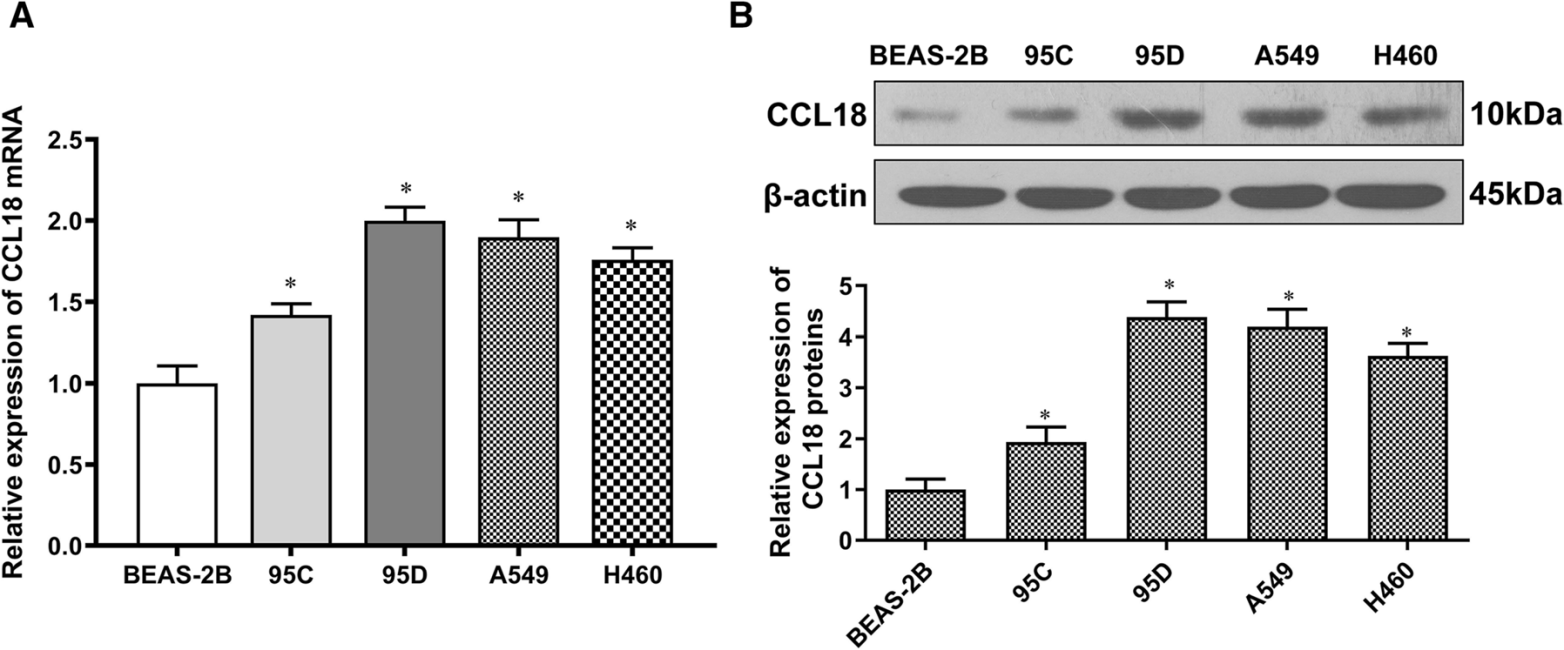
CCL18 expression in NSCLC cells. **A** RT-qPCR analysis of CCL18 mRNA expression in human normal lung epithelial cell line (BEAS-2B) and NSCLC cell lines. **B** Western blot analysis of CCL18 protein expression in human normal lung epithelial cell line (BEAS-2B) and NSCLC cell lines. *β*-actin was a loading control. *, *p* < 0.05 compared to 2B group

According to our previous study, CCL18 was not only highly expressed in tumor tissues and the serum of NSCLC patients but also significantly associated with a poor prognosis [17]. Herein, we explored whether CCL18 played a role in regulating in vitro growth of NSCLC cells. To this end, we initially examined the expression of CCL18 in 95C, 95D, A549, and H460 lung cancer cell lines. According to our results (Fig. 1A), the mRNA levels of CCL18 were significantly increased in lung cancer cells relative to the normal lung epithelial cells. The elevated expression of CCL18 protein lung cancer cells was further confirmed by Western blot analysis (Fig. 1B). Of note, the expression level of CCL18 was significantly higher in the high-metastatic lung cancer cells 95D than that in the low-metastatic lung cancer cells 95C, suggesting that CCL18 expression might be closely related to the invasion and metastasis capability of lung cancer cells.

### Effects of omega-3 on the metastasis and CCL18 expression of NSCLC cells

**Fig. 2:**
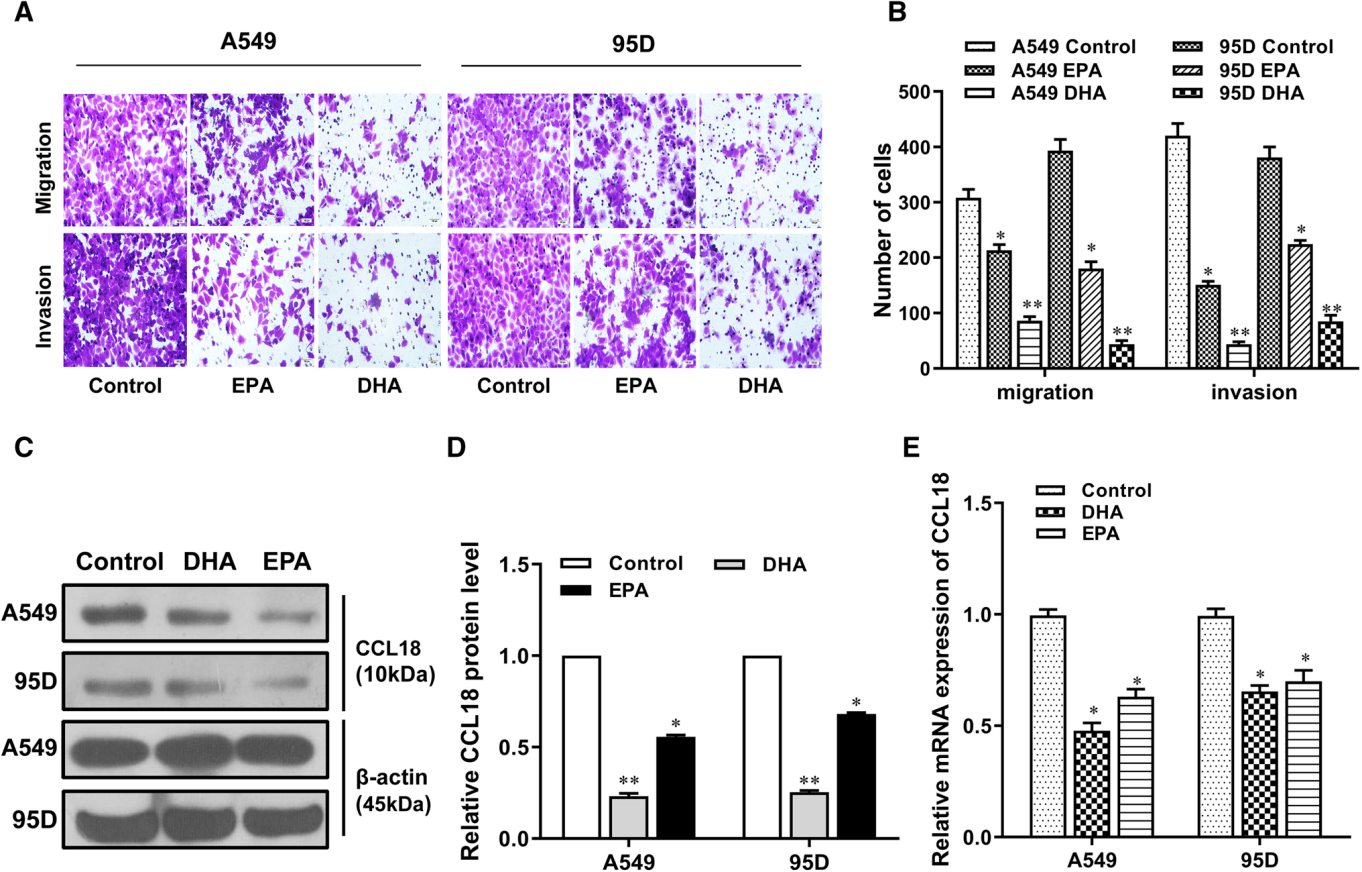
Effects of different types of omega-3 on NSCLC cells. A549 and 59D cells were exposed to EPA and DHA for 48 h. Metastasis and invasion abilities were analyzed by Transwell assays (scale bar: 100 µm) (**A**–**B**). CCL18 protein expression was assessed by Western blot (**C**–**D**) and CCL18 mRNA expression was assessed by RT-qPCR (**E**). *, *p* < 0.05; **, *p* < 0.01 compared to control group

To determine the effect of omega-3 on lung cancer cell metastasis, we selected two major omega-3 DHA and EPA to treat NSCLC cell lines, A549 and 95D. Our results indicated that both DHA and EPA could obviously suppress the migration and invasion abilities of A549 and 95D cells, but the inhibitory effect of DHA was significantly stronger than that of EPA (Fig. 2A and B). Meanwhile, we found that treatment with DHA and EPA robustly reduced CCL18 expression in A549 and 95D lung cancer cells, whereas the inhibitory effect of DHA was also more pronounced than that of EPA (Fig. 2C–E). These data indicate that omega-3, especially DHA, inhibited NSCLC cell metastasis possibly by downregulating CCL18 expression. Therefore, we chose DHA as a treatment factor for subsequent experiments.

### DHA inhibited the migration and invasion of NSCLC cells

**Fig. 3:**
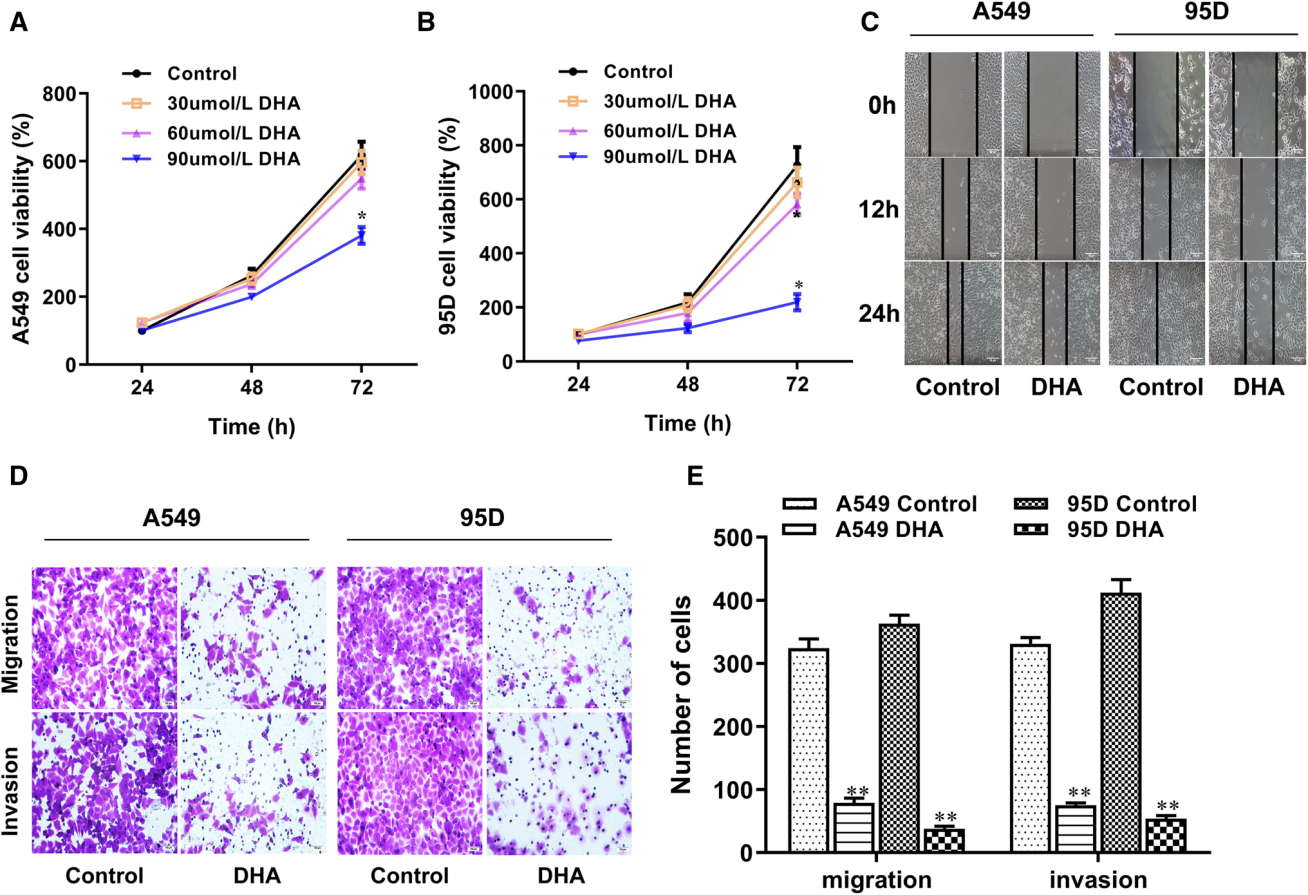
DHA inhibited the metastasis and invasion in A549 and 95D cells. **A**–**B** Cell viability of A549 and 95D cells treated with DHA at different concentrations and times were analyzed by CCK-8 assays. Cell metastasis and invasion were assessed by wound healing (scale bar: 50 µm) **C** and Transwell assays (scale bar: 100 µm) (**D**–**E**) in A549 and 95D cells exposed to 60 μmol/L DHA. *, *p* < 0.05 compared to control group

To comprehensively explore the effect of DHA on the invasion and migration of lung cancer cells, we first examined the effect of different concentrations of DHA on the growth and proliferation of A549 and 95D lung cancer cells by CCK8 assay and found that DHA at both 30 μmol/L and 60 μmol/L DHA had no obvious effect on the proliferation of lung cancer cells, whereby only at a relatively high concentration, 90 μmol/L, DHA significantly inhibited the proliferation of lung cancer cells (Fig. 3A and B). To rule out the possibility that DHA inhibits migration and metastasis of lung cancer cells due to its inhibitory effect on cell proliferation, we then chose 60 μmol/L as the working concentration of DHA for the subsequent experiments. According to in vitro scratch wound healing assay, the presence of DHA for 48 h remarkably attenuated the migration ability of NSCLC cells (Fig. 3C). Transwell assays also indicated that there was obviously less invading or migrating cells in the lower chamber in DHA-treated group than the control (Fig. 3D). These results implied that DHA might deter or suppress NSCLC progression by attenuating the migration and invasion capability of cancer cells.

**Fig. 4:**
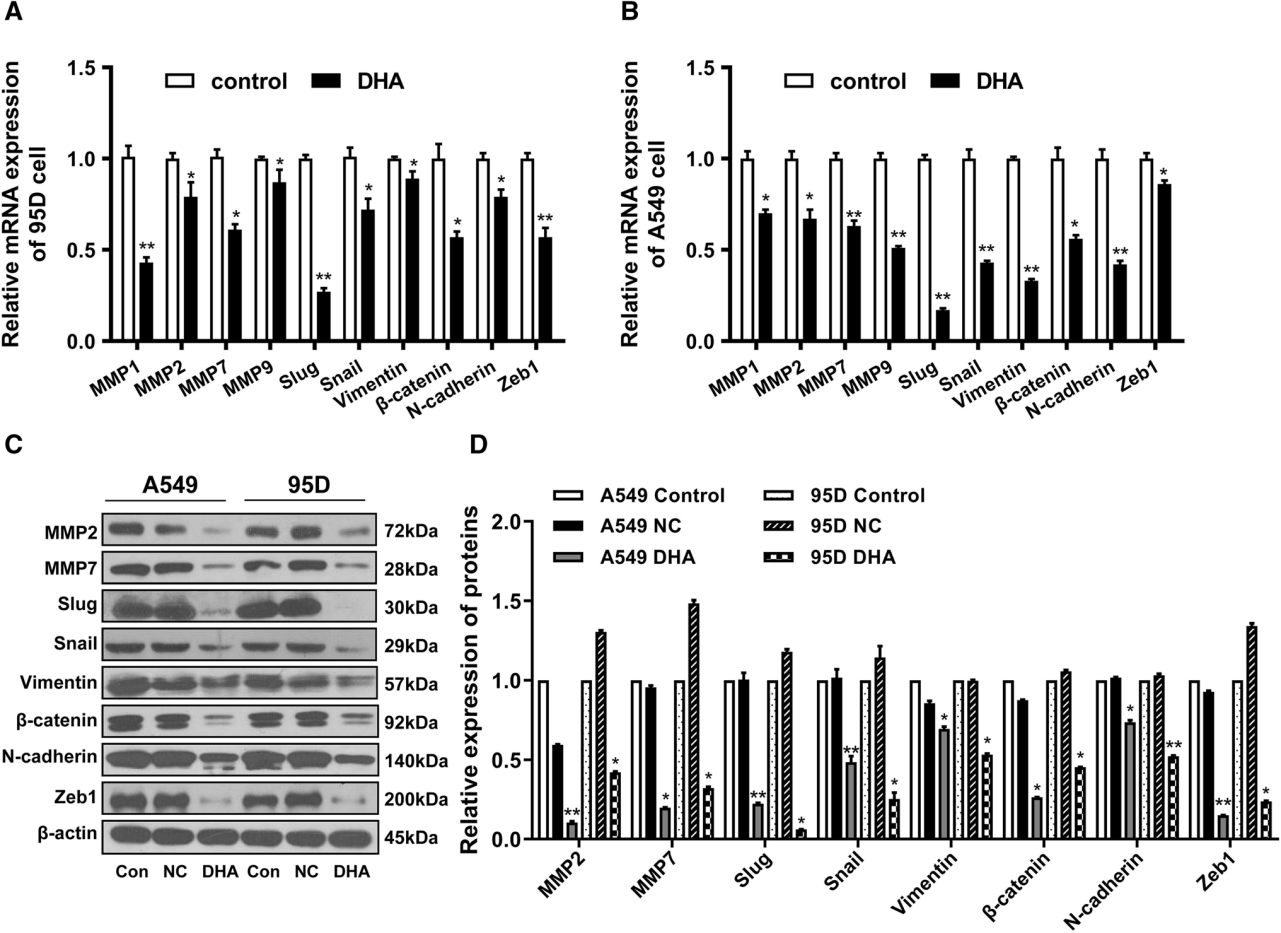
DHA suppressed the expression of metastasis-related genes. **A**–**B** Metastasis-related mRNA expression of A549 and 95D cells treated with DHA were analyzed using RT-qPCR. **C**–**D** Metastasis- and EMT-related proteins expression of A549 and 95D cells treated with DHA were assessed using Western blot. *, *p* < 0.05; **, *p* < 0.01 compared to control group

In addition, we determined the changes in the expression of metastasis- and EMT-related genes in DHA-treated NSCLC cells. Our results indicated that DHA treatment remarkably decreased the mRNA expression levels of metastasis-associated enzymes, MMP2 and MMP7 (Fig. 4A and B). Notably, the expression of EMT-related marker genes, such as vimentin, *β*-catenin, N-cadherin, snail, slug, and zeb1, was significantly decreased in DHA-treated cells compared with that in controls (Fig. 4C and D). These results suggest that DHA inhibited invasion and migration of NSCLC cells possibly by inhibiting or reversing the EMT process.

### CCL18 rescued the DHA-induced inhibition of NSCLC cell metastasis

**Fig. 5:**
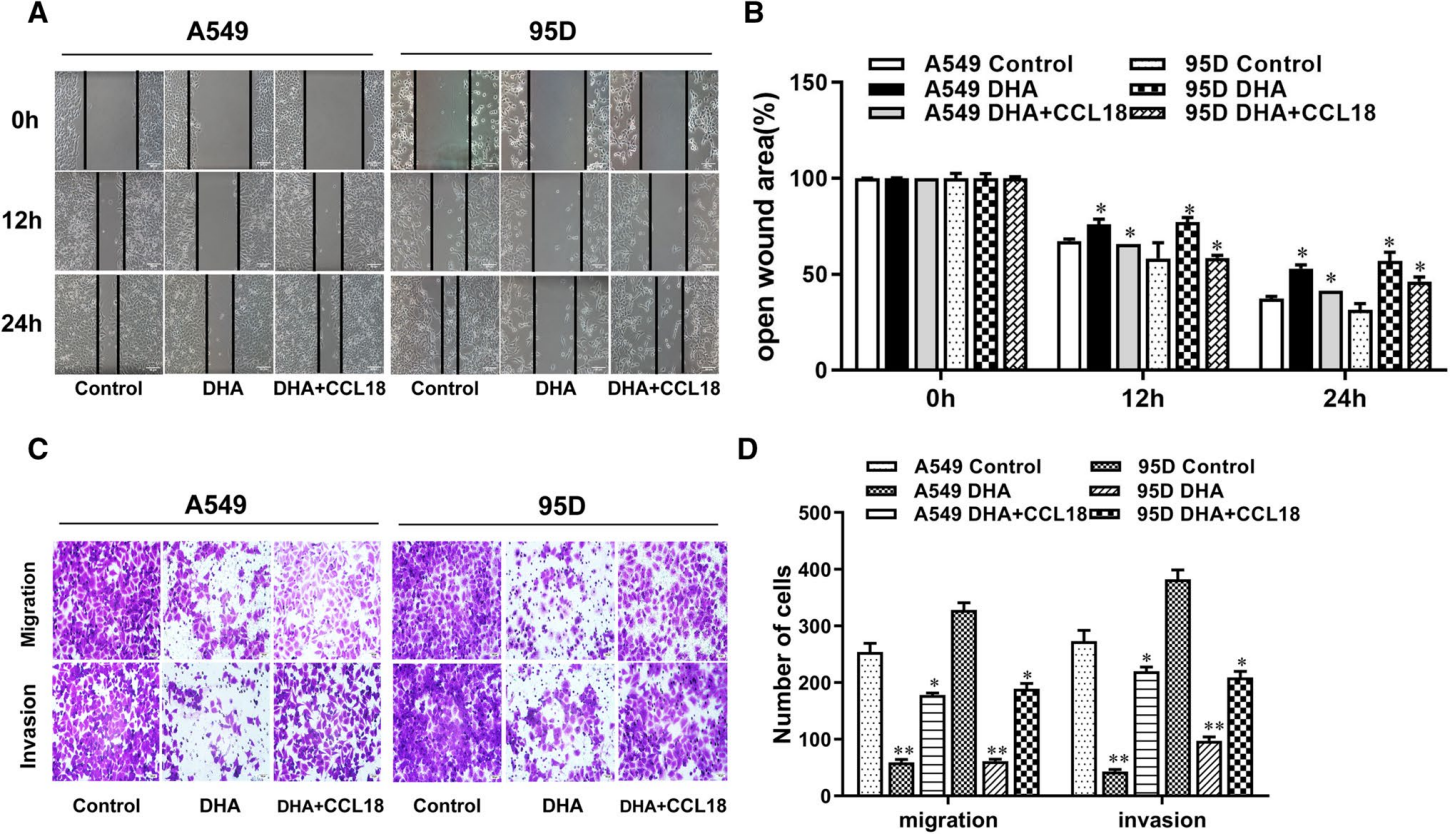
CCL18 rescued DHA-mediated suppression of metastasis and invasion abilities in lung cancer cell. A549 and 95D cells were treated with DHA in the presence or absence of CCL18(10 ng/mL). Cell metastasis and invasion were analyzed by wound healing (scale bar: 50 µm) (**A**–**B**) and Transwell assays (scale bar: 100 µm) (**C**–**D**). *, *p* < 0.05 compared to control group

**Fig. 6:**
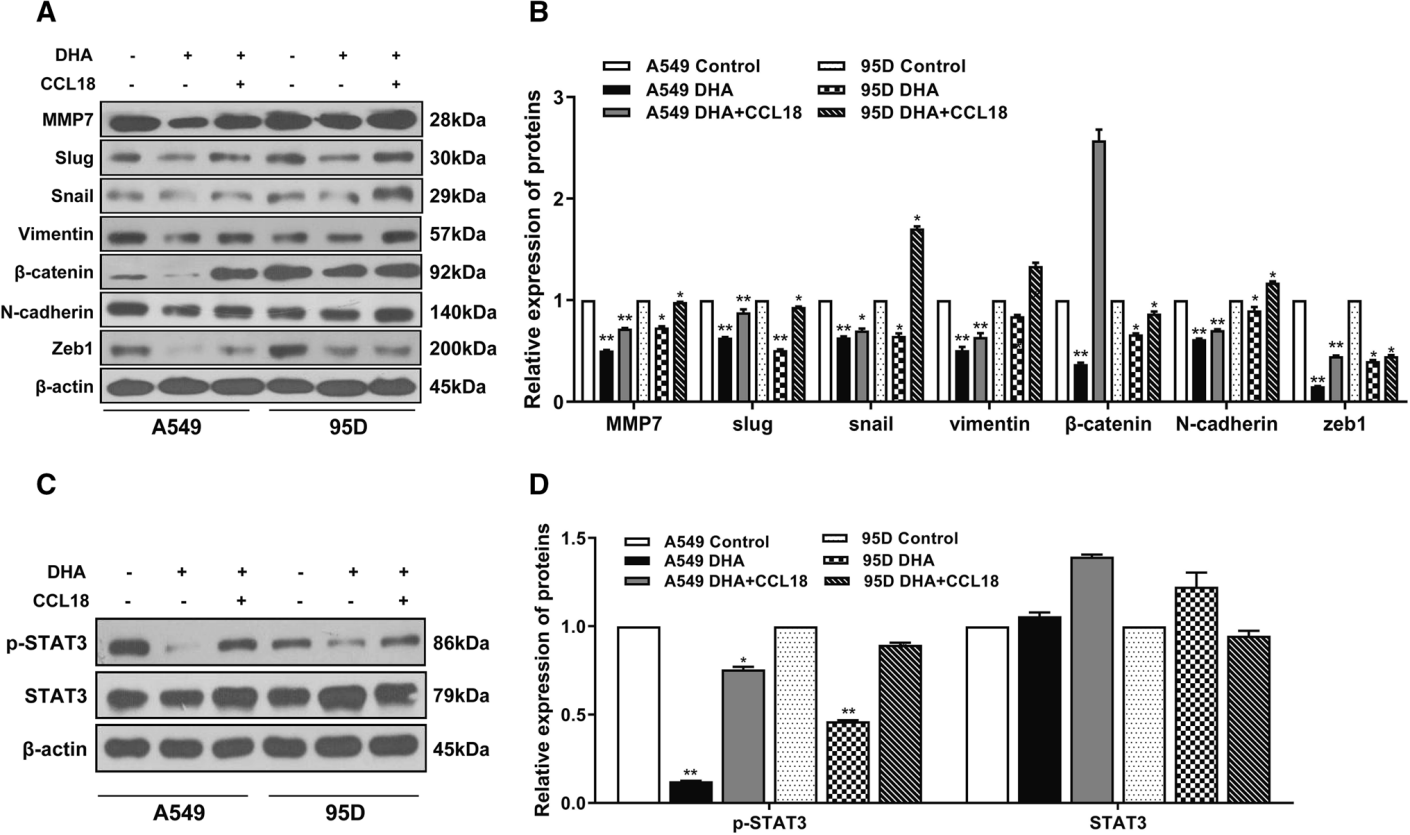
CCL18 attenuated DHA-mediated downregulation of metastasis-related proteins. A549 and 95D cells were treated with DHA in the presence or absence of CCL18(10 ng/mL). Metastasis- and EMT-related proteins (**A**–**B**) and p-STAT3 (**C**–**D**) expression were assessed using Western blot. *, *p* < 0.05; **, *p* < 0.01 compared to control group

Given that DHA significantly inhibited cellular CCL18 expression, and simultaneously, the invasion and migration capability of NSCLC cells, we then determined whether downregulation of CCL18 expression is directly involved in DHA-mediated suppression of lung cancer cell metastasis. To this purpose, both A549 and 95D lung cancer cells were treated with DHA in the presence or absence of exogenous CCL18, and then, cell migration and invasion of lung cancer cells were evaluated by in vitro wound healing and transwell assay, respectively. According to our results, the presence of exogenous CCL18 efficiently reversed DHA-mediated inhibition of migration and invasion in NSCLC cells (Fig. 5A–D). Accordingly, Western blot analysis revealed that exogenous CCL18 attenuated DHA-mediated downregulation in the expression of metastasis- and EMT-related genes, including MMP7, Snail, Slug, Vimentin, *β*-catenin, N-cadherin, and Zeb1, in NSCLC cells (Fig. 6A and B), further supporting the notion that DHA inhibits NSCLC cell metastasis possibly due to the downregulated CCL18 expression. A recent study has shown that stimulation of oral cancer cells with CCL18 led to the activation of STAT3 signaling pathway that plays an important role in the growth and EMT process in cancer [18]. Our results indicated that DHA treatment effectively decreased the level p-STAT3, the active form of STAT3, in lung cancer cells but had no effect on the level of the total STAT3 (Fig. 6C and D). As expected, the presence of CCL18 rescued DHA-mediated downregulation of p-STAT3 (Fig. 6C and D). Altogether, these findings suggest that DHA suppresses migration and metastasis possibly by inhibiting EMT process via interfering with CCL18/STAT3 pathway in NSCLC cells.

## Discussion

In the complex tumor microenvironment (TME), tumor cells dynamically interact with various types of noncancerous cells and noncellular components that play essential roles in the clonal selection, heterogeneity, proliferation, metastasis, and therapeutic resistance of cancer cells [19]. Chemotactic cytokines or chemokines in TME play pivotal roles in regulating the migration, recruitment, and interaction of different subtypes of cells with either pro- or antitumor function [20]. CCL18, a chemokine mainly secreted by macrophages and dendritic cells, has been shown to play important roles in the reprogramming of TME by the recruitment of immune cells and direct regulation of multiple functions of tumor cells, such as cell proliferation, EMT, and metastasis [19, 20]. Our previous study showed that CCL18 was highly expressed in tumor tissues and the serum and was also closely associated with lymph node metastasis and a poor prognosis in NSCLC patients [16], suggesting that CCL18 plays an important role in NSCLC progression, especially in tumor metastasis. Therefore, CCL18 might be a novel molecular target for NSCLC therapy.

Surgery, chemo- and radiation-therapies remain the mainstays of cancer treatment. Due to the serious side effects of traditional radiotherapy and chemotherapy, nutritional interventions are emerging as a novel intervening approach for tumor therapy. Omega-3 fatty acids, mainly including DHA and EPA, are naturally non-toxic compounds serving as the important essential nutrients in the body. Accumulating evidence has shown that omega-3 has potent inhibitory effects on tumorigenesis and progression in a variety of tumors [21]. For instance, previous studies have shown that the application of omega-3 in colon cancer patient promoted the response rates and improved the quality of life without enhancing toxicity [22]. Likewise, our previous study also showed that omega-3 metabolites protected against CXCR4-associated melanoma metastasis [23], indicating that omega-3-mediated inhibition of tumor metastasis may be associated with the reprogramming of TME. Meanwhile, we also found that both the serum tumor biomarkers and CCL18 levels of lung cancer patients were significantly reduced after two weeks of dietary omega-3 intake(the data no shown), thus further supporting the notion that omega-3 exerts the antitumor effects possibly by inhibiting CCL18 expression. However, the related mechanisms remain largely unknown.

In the present study, we found that the two major omega-3 EPA and DHA at 60 µM had no obvious effect on the growth and proliferation of NSCLC cells, but effectively inhibited the migration and invasion of lung cancer cells, whereby the inhibitory effect of DHA was more pronounced that of EPA. Recently, Goupille et al. [24] reported that the levels of omega-3 in breast adipose tissue were negatively correlated with the development of bone metastases in premenopausal women with breast cancer. Pfister et al. [25] found that omega-3 suppresses the invasion in colorectal cancer cells by a PI3K-dependent mechanism but had no effect on cell proliferation. In addition, in vivo studies in preclinical animal models also showed that oral supplementation of EPA/DHA significantly decreased tumor growth and metastatic progression in TNBC mice [26]. Therefore, omega-3 fatty acid treatment is emerging as an important adjuvant in combination with chemotherapy so as to reduce toxicity and resistance of chemotherapy and inhibit metastasis in cancer [27]. In the present study, our results showed that omega-3, especially DHA, remarkably suppressed the migration and invasion capability of lung cancer cells together with a decreased CCL18 expression in NSCLC A549 and 95D cells, indicating that omega-3 (especially DHA) effectively suppress the migration and invasion of NSCLC cells possibly by downregulating CCL18 expression.

EMT is a plastic process in which epithelial cells lose their polarized organization and acquire migratory and invasive mesenchymal features, thus contributing to invasion and metastasis of tumor cells [28]. In this study, we found that DHA not only markedly decreased the mRNA and protein expression levels of metastasis-associated enzymes, such as MMP1, MMP2, MMP7 and MMP9, but also significantly downregulated the expression of EMT-related or regulatory genes, such as vimentin, *β*-catenin, N-cadherin, Snail, Slug, and Zeb1, in NSCLC cells. Additionally, we showed that the presence of exogenous CCL18 robustly revered DHA-mediated inhibition of NSCLC cell migration and invasion. These findings support the notion DHA inhibits EMT and metastasis by directly downregulating CCL18 expression in NSCLC cells.

JAK/STAT3 is one of the important signaling pathways that play pivotal roles in the regulation of cell proliferation, EMT process, cancer stem cell properties, metastasis, and chemoresistance in a variety type of cancer [29]. Most recently, it has been shown that stimulation with CCL18 activated STAT3 signaling pathway in oral cancer cells [18]. In the present study, we showed that DHA treatment dramatically decreased p-STAT3 levels, the activated form of STAT3, in NSCLC cells, which was remarkably rescued by exogenous CCL18, thereby suggesting that DHA inhibits EMT and metastasis possibly by interfering with CCL18/STAT3 signaling pathway in NSCLC cells. However, mechanistic studies in detail are warranted in our future study.

## Conclusions

In summary, our findings have demonstrated that omega-3, especially DHA, can effectively suppress the migration and invasion of NSCLC cells possibly by inhibiting EMT process through interfering with CCL18/STAT3 signaling pathway. These findings have shed light on the potential application of omega-3 as an adjuvant therapy for metastatic NSCLC.

### Supplementary Information

Below is the link to the electronic supplementary material.

Supplementary file1 (PDF 2322 kb)

Supplementary file2 (PDF 2346 kb)

## Abbreviations

NSCLC: Non-small cell lung cancer
CCL18: Chemokine ligand 18
EMT: Epithelial-mesenchymal transition
DHA: Docosahexaenoic acid
TAM: Tumor-associated macrophages
ALA: Alpha-linolenic acid
EPA: Eicosapentaenoic acid
MMPs: Matrix metalloproteinases
LSD: Least-significant difference
