# Supplementary material for: DHA inhibits invasion and metastasis in NSCLC cells by interfering with CCL18/STAT3 signaling pathway
Source: Clin Exp Med. 2022 Oct 10;23(6):2311–20. doi: 10.1007/s10238-022-00906-0 (PMC10543829; doi:10.1007/s10238-022-00906-0)
Supplement: Supplementary file 2 — Supplementary file2 (PDF 2346 kb) [file 10238_2022_906_MOESM2_ESM.pdf]

Cell Line Authentication Service
STR Profile Report

**Sample Submitted By:**	Dr. Xudong Tang
	Guangdong Medical University
**Email Address:**	txd@gdmu.edu.cn tangxudong2599@126.com
**Sales Order:**	220828F
**Cell Line Designation:**	A549
**Date Sample Received:**	Aug 28th, 2022
**Report Date:**	Aug 30th, 2022

Methodology: Twenty-one short tandem repeat (STR) loci, one Y-Indel locus plus the Amelogenin locus were amplified using the commercially available SiFaSTR™ 23 plex Kit. The cell line sample was processed using the ABI Prism® 3130 XL Genetic Analyzer. Data were analyzed using GeneMapper® ID v3.2 software (Applied Biosystems). Appropriate positive and negative controls were run and confirmed for each sample submitted.

Data Interpretation: Cell lines were authenticated using Short Tandem Repeat (STR) analysis as described in 2012 in ANSI Standard (ASN-0002) by the ATCC Standards Development Organization (SDO) and in Capes-Davis et al., Match criteria for human cell line authentication: Where do we draw the line? Int J Cancer. 2013;132(11):2510-9.

GTB™ performs STR Profiling following ISO 9001:2008 and ISO/IEC 17025:2005 quality standards.

There are no warranties with respect to the services or results supplied, express or implied, including, without limitation, any implied warranty of merchantability or fitness for a particular purpose. Genetic Testing Biotechnology (GTB) is not liable for any damages or injuries resulting from receipt and/or improper, inappropriate, negligent or other wrongful use of the test results supplied, and/or from misidentification, misrepresentation, or lack of accuracy of those results. Your exclusive remedy against GTB and those supplying materials used in the services for any losses or damage of any kind whatsoever, whether in contract, tort, or otherwise, shall be, at GTB's option, refund of the fee paid for such service or repeat of the service.

NOTE: According to the recommendations of *IJC* on cell line authentication, the report is valid for 3 years since the issue date.

Technical Questions?
GTB Technical Support
+86-512-67486171
service@jsdna.org
Section 505, Yixin BLD
SIP, Suzhou, 215123
Jiangsu, P.R. China

Ordering Questions?
order@jsdna.org
GTB Corporation
+86-512-62806339
Section 303, Yixin BLD
SIP, Suzhou, 215123
Jiangsu, P.R. China

GENETIC TESTING BIOTECHNOLOGY

Cell Line Authentication Service
STR Profile Report

Sales Order: 220828F

	Test Results for Submitted Sample		ExPASy Reference Database Profile	
**Loci**	**Query Profile:** A549		**Database Profile:** Hela.P3	
Amelogenin	X		X	
D3S1358	15	18		
D5S818	11	12	11	12
D2S1338	17			
TPOX	12		12	
CSF1PO	9	10	9	10
Penta D	8	15		
Y- Indel	-			
TH01	7		7	
vWA	16	18	16	18
D7S820	8	12	8	12
D21S11	27	28		
Penta E	7	17		
D10S1248	13	15		
D8S1179	13			
D1S1656	12	15		
D18S51	16			
D12S391	20	25		
D6S1043	18			
D19S433	13	14		
D16S539	9	10	9	10
D13S317	12	13.3	12	13.3
FGA	18	21		

The allele match algorithm compares the 8 core loci plus amelogenin only, even though alleles from all loci will be reported when available.

Note: Loci highlighted in grey (8 core STR loci plus Amelogenin) can be made public to verify cell identity. In order to protect the identity of the donor, ***please do not publish*** *the allele calls from all the STR loci tested.*

The sample match is based on the reference data available at the time of comparison.

Explanation of Test Results

Cell lines with ≥80% match are considered to be related; i.e., derived from a common ancestry. Cell lines with between a 55% to 80% match require further profiling for authentication of relatedness.

☐ The submitted sample profile is human, but not a match for any profile in the ExPASy STR database.

☑ The submitted profile is an exact match for the following human cell line(s) in the ExPASy STR database (8 core loci plus Amelogenin): Hela.P3

☐ The submitted profile is similar to the following ExPASy human cell line(s):

e-Signature Technician: Digitally signed by Xuekun Chen
DN: cn=Xuekun Chen, o=Genetic Testing Biotechnology (Suzhou), ou=DNA Typing Section, email=order@jsdna.org, c=CN
Date: 2022.08.30 16:11:30 +08'00'

e-Signature Reviewer: Digitally signed by Xiankun Zhao
DN: cn=Xiankun Zhao, o=Genetic Testing Biotechnology (Suzhou), ou=Supervision Section, email=service@jsdna.org, c=CN
Date: 2022.08.30 16:11:56 +08'00'

GENETIC TESTING BIOTECHNOLOGY · GUARANTEE · CELL LINE AUTHENTICATION

More information

Addendum: Electropherogram for the customer's sample set 1 of 1

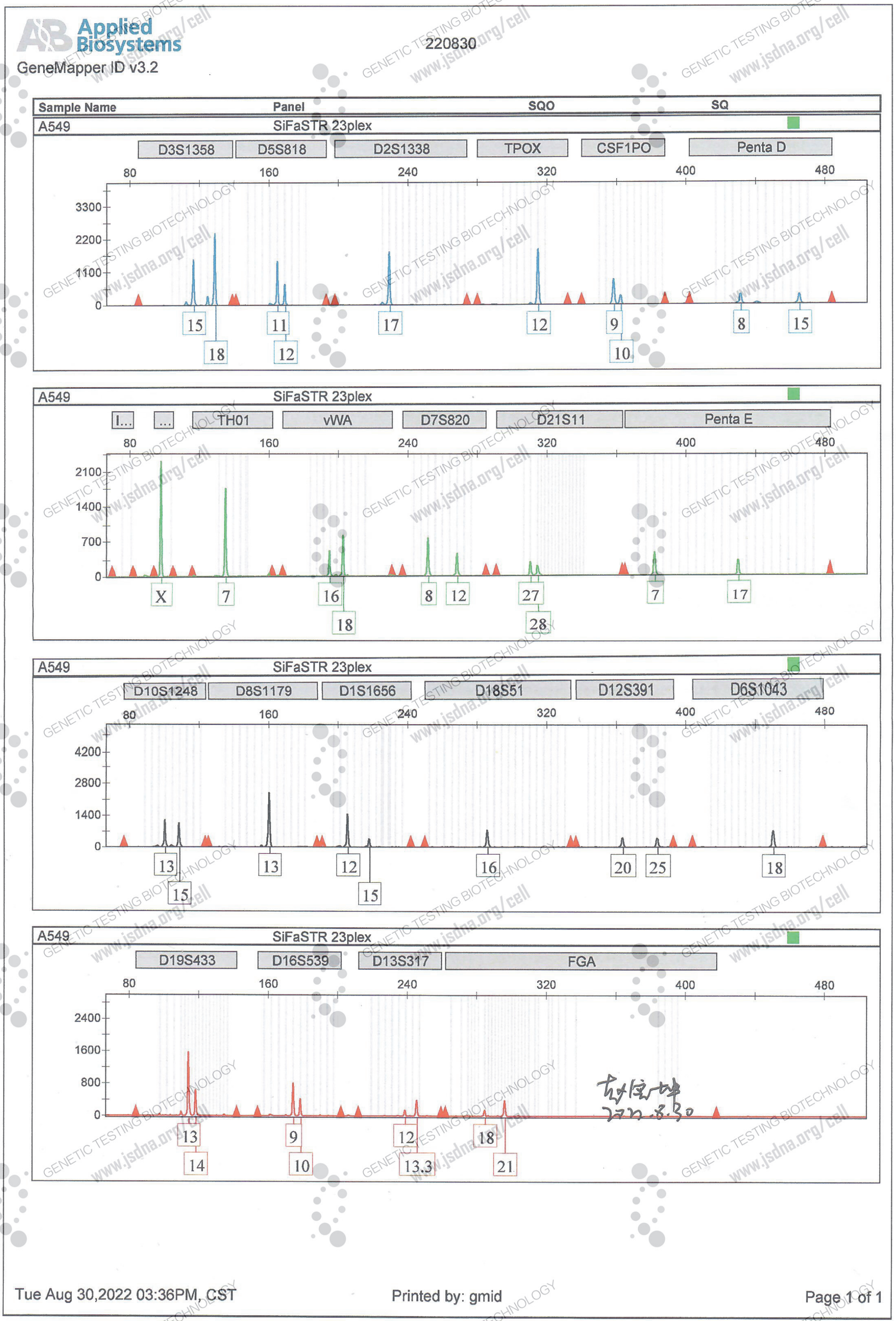
Applied Biosystems
GeneMapper ID v3.2
220830
Sample Name | Panel | SQO | SQ
A549 | SiFaSTR 23plex
D3S1358 D5S818 D2S1338 TPOX CSF1PO Penta D
15 18 11 12 17 12 9 10 8 15
A549 | SiFaSTR 23plex
TH01 vWA D7S820 D21S11 Penta E
X 7 16 18 8 12 27 28 7 17
A549 | SiFaSTR 23plex
D10S1248 D8S1179 D1S1656 D18S51 D12S391 D6S1043
13 15 13 12 15 16 20 25 18
A549 | SiFaSTR 23plex
D19S433 D16S539 D13S317 FGA
13 14 9 10 12 13.3 18 21
Tue Aug 30,2022 03:36PM, CST
Printed by: gmid
Page 1 of 1
